# Mechanical Properties of Thermoplastic Polymers for Aligner Manufacturing: In Vitro Study

**DOI:** 10.3390/dj8020047

**Published:** 2020-05-10

**Authors:** Francesco Tamburrino, Vincenzo D’Antò, Rosaria Bucci, Giulio Alessandri-Bonetti, Sandro Barone, Armando Viviano Razionale

**Affiliations:** 1 DICI—Department of Civil and Industrial Engineering, Università di Pisa, 56122 Pisa, Italy; sandro.barone@unipi.it (S.B.); a.razionale@ing.unipi.it (A.V.R.); 2 Department of Neurosciences, Reproductive Sciences and Oral Sciences, Università di Napoli Federico II, 80131 Napoli, Italy; vincenzo.danto@unina.it (V.D.); rosaria.bucci@unina.it (R.B.); 3 Unit of Orthodontics, Department of Biomedical and Neuromotor Sciences (DIBINEM), Università di Bologna, 40125 Bologna, Italy; giulio.alessandri@unibo.it

**Keywords:** orthodontic aligners, thermoplastic polymers, mechanical properties, simulated oral environment

## Abstract

The use of metal-free thermoplastic materials plays a key role in the orthodontic digital workflow due to the increasing demand for clear aligner treatments. Three thermoplastic polymers commonly used to fabricate clear aligners, namely Duran^®^, Biolon^®^ and Zendura^®^, were investigated to evaluate the effect of thermoforming (T.), storage in artificial saliva (S.A.S.) and their combination on their mechanical properties. Elastic modulus and yield stress of the specimens were characterized. Each material was characterized for each condition through tensile tests (ISO527-1). The results showed that thermoforming does not lead to a significant decrease in yield stress, except for Zendura^®^ that showed about a 30% decrease. An increase of the elastic modulus of Duran^®^ and Zendura^®^, instead, was observed after thermoforming. The same increase was noticed for the yield stress of Duran^®^. For S.A.S. specimens, the elastic modulus generally decreases compared to supplier condition (A.S.) and simply thermoformed material. A decrease of yield stress, instead, is significant for Zendura^®^. The results demonstrated that the impact of the operating conditions on the mechanical properties can vary according to the specific polymer. To design reliable and effective orthodontic treatments, the materials should be selected after their mechanical properties are characterized in the simulated intraoral environment.

## 1. Introduction

Adolescent and adult patients who are aware of their malocclusion traits and dissatisfied with their dental appearance tend to have psychosocial concerns [1,2]. On the other hand, the use of an orthodontic appliance to treat a malocclusion may lead to an impairment of quality of life, which is less severe when clear aligner therapy is performed [3]. Despite the fact that orthodontic treatment with clear aligners is a quickly growing sector, there is still insufficient evidence with regard to the effectiveness and stability of the treatment compared with conventional brackets [4]. Aligner therapy might treat mild non-extraction cases faster and more efficiently, but it requires more time than fixed appliance treatment for more complex cases. Furthermore, there is still limited evidence of aligner efficacy in arch expansion through bodily tooth movement, extraction space closure, and larger antero-posterior and vertical discrepancies [5].

The two main domains for implementing reliable protocols to treat malocclusions according to diagnosis are the development of a proper staging and the study of biomechanics in order to deliver desired force systems [6]. Therefore, the mechanical performance of thermoplastic material used for aligners plays a critical role in obtaining desired results in difficult orthodontic movements [7], together with aspects such as aligner thickness, geometry design and control, which are also deemed critical in order to get a proper tooth-aligner system [8,9,10]. In particular, the orthodontic thermoplastic materials should have specific characteristics including biocompatibility, transparency, low hardness, good elasticity, resilience and resistance to storage in artificial saliva [11]. Since the mechanical properties of the materials used for production of clear aligners are of utmost importance to evaluate the efficacy of an orthodontic treatment based on these devices, the technical data provided by materials’ suppliers cannot always be used as a reference, but the materials need to be experimentally assessed within the different conditions in which they are used. Mechanical properties of thermoplastic polymers can change after thermoforming, suggesting the need to test them after this procedure [12]. Furthermore, in the oral cavity, aligners are subjected to an aggressive environment that could lead to a high degradation of their properties causing a negative influence on treatment efficacy [13]. The aim of this study is to evaluate the effect of the thermoforming process and of storage in artificial saliva on the mechanical properties of three different thermoplastic materials, commonly used for clear aligners, or rather Duran^®^, Biolon^®^ and Zendura^®^. Duran^®^, is designated by the manufacturer as PETG. It is a clear amorphous copolymer of polyethylene terephthalate (PET), with good mechanical properties, formability, optical qualities, fatigue resistance, and dimensional stability [14]. It is characterized by a low level of hygroscopy and a good manufacturability, since, for thermoforming, pre-drying is not normally required. Biolon^®^, instead, is designated by the manufacturer as PET. It is a PETG that is not glycol modified, normally semi-crystalline, and, according to the degree of crystallinity, has mechanical properties that can change. Finally, Zendura^®^ is designated as TPU. It is one of the most versatile engineering thermoplastics, highly abrasion resistant and elastic, with a high shear strength and a good transparency [11,15]. TPU is also characterized by a two-phase microstructure consisting of hard and soft segments, where the latter tends to be oriented perpendicular to the applied stresses, and then breaks into smaller pieces allowing further deformation [16]. The specimens of these materials have been characterized through a set of tensile tests carried out under different conditions. The tests have the purpose of analyzing the effect that the manufacturing process, and subsequently the operating environment of the aligner, may have on the mechanical properties of the material. For this reason, the specimens were subjected to thermoforming and subsequent storage in a chemical solution, reproducing the biochemical environment of human saliva. For each material and condition, the elastic modulus and yield stress were characterized and the relative data sets were analyzed to verify the significance of the statistical difference through a two-sample t-test. Finally, representative stress-strain curves were reported and the mechanical behavior analyzed.

## 2. Materials and Methods

**Figure 1:**
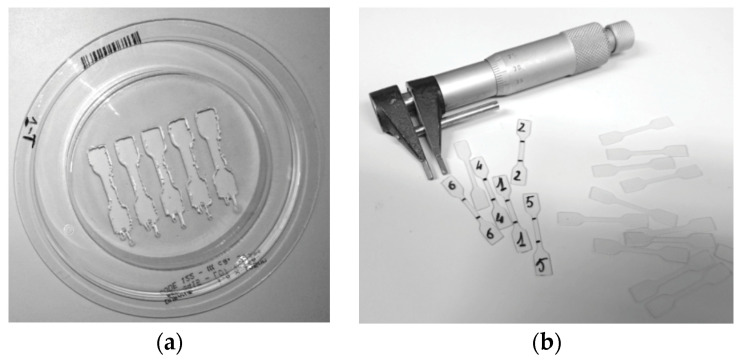
Thermoformed material after waterjet cutting (**a**) and resulting specimens (**b**).

**Figure 2:**
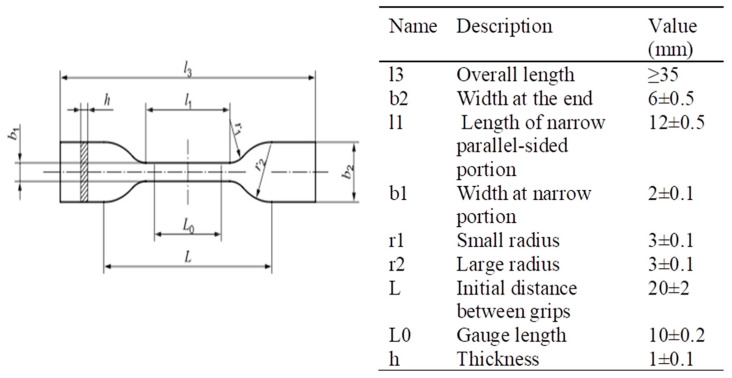
Specimen geometry and dimensions.

The experimental tests were designed on the EN ISO 527-1:2012 [17], which specifies general principles, conditions and procedures for determining tensile properties of plastic materials. The materials investigated were: Duran^®^ supplied by Scheu Dental GmbH (Iserlohn, Germany), Biolon^®^ supplied by Dreve Dentamid GmbH (Unna, Germany) and Zendura^®^ supplied by ZenduraDental (Lakeview Blvd, Fremont, CA, USA). For each material we carried out tests on material that was not yet thermoformed (as from supplier, A.S.), after the thermoforming process (no storage in artificial saliva) and after thermoforming process plus storage in artificial saliva. The procedure was designed as described to take into account the influence of manufacturing process and the possible effect of material degradation due to a simulated intraoral environment. All materials were supplied as foils with a circular shape (see Figure 1) with a diameter of 125 mm and a thickness of 0.75 or 1 mm. For the specimen preparation of all the materials investigated a thermoforming process was performed under manufacturers’ recommendations for pressure, heating and cooling time and according to the procedure normally adopted to manufacture clear aligners. The thermoforming machine used a Ministar S^®^, provided by Scheu-Dental^®^. With this machine the side of the material, which is placed directly over the model (i.e., the model acting as positive mold), is heated by a short-wave, thermally controlled infrared heater. The machine allows one to select heating and cooling time and the pressure to apply over the heated foil of material. The parameters used for the thermoforming process of each material are reported in Table 1. In the present study a 3D printed disk of 80 mm diameter and 12 mm height was used as a mold for the thermoforming machine. The geometry of the mold was designed to provide a flat area where the material could be thermoformed and the specimens could be cut. The specimens were cut according to shape, size and thickness suggested by the normative EN ISO 527-2, specimen type 5B (see Figure 2) [14,17]. For specimen cutting, a high pressure waterjet and a manual refinement were used (see Figure 1). Then, the cross section dimensions of each specimen were accurately measured with a micrometer (CLM1-15QM, Mitutoyo Co.). Treatment based on the storage in artificial saliva was programmed to reproduce the influence of the saliva corrosion on the aligner, through the use of an artificial mixture aimed to recreate the biochemical environment of human saliva [16,18]. The chemical composition of the artificial saliva used is reported in Table 2.

The specimens were first weighed to evaluate the quantity of fluid absorbed after the storage in artificial saliva (see Table 3). The treatment consisted of a bath of the specimens in the prepared compound (as in Table 2) for 7 days at a temperature of 37 °C. This time corresponds to half of the time of use of one aligner during the actual orthodontic treatment. Nevertheless, it is justified because the fluid absorption from plastic materials occurs mainly in the first 72–168 h as discussed in [18,19,20].

**Figure 3:**
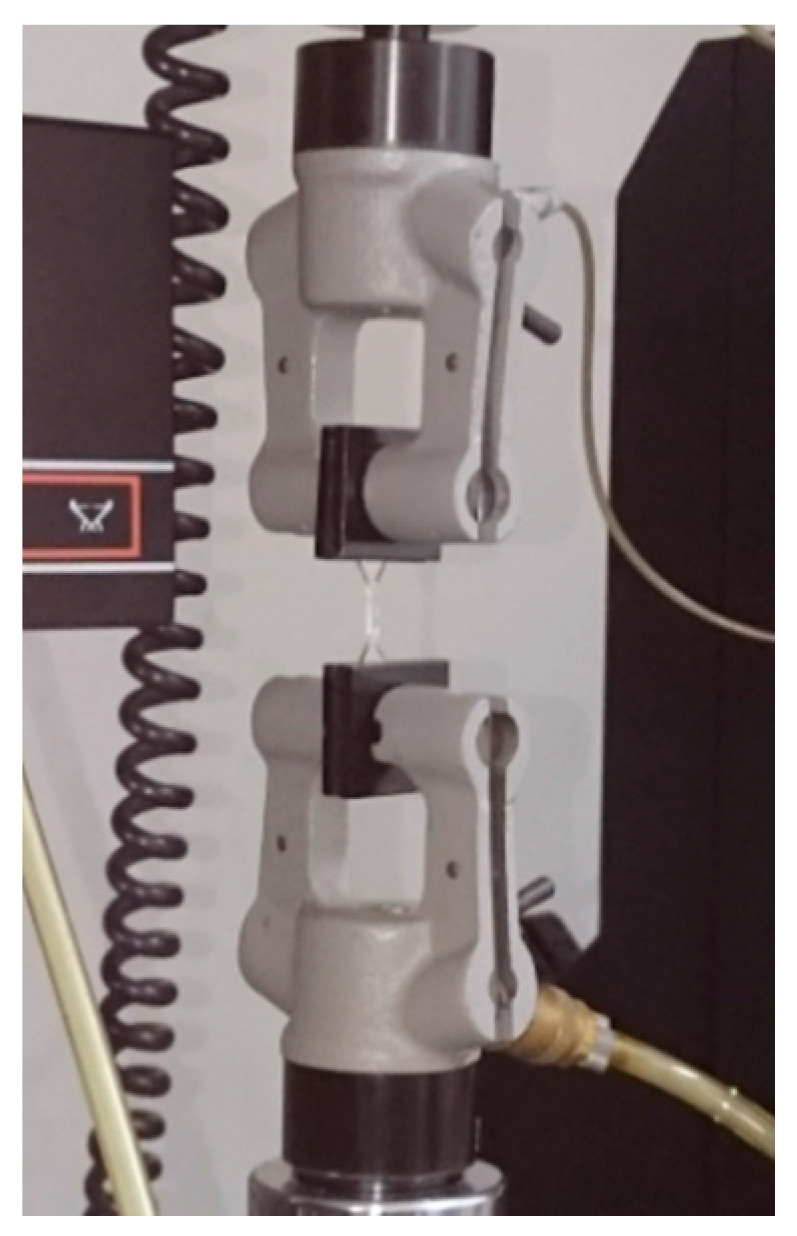
Specimen clamping during tensile test by Universal Instron Testing Machine 5500R.

At the end of the storage in artificial saliva, the weight variation was evaluated for establishing the quantity of fluid absorbed. Finally, the tensile tests, for each material condition investigated (before thermoforming, after the thermoforming and after thermoforming plus storage in artificial saliva), were performed on the specimens by the Instron Universal Testing Machine 5500R (see Figure 3) at a temperature of 23 °C. The measurement and the online monitoring of the strain were performed through the use of an optical system. The experimental tests were designed for determining the elastic modulus and the tensile yield stress. To correspond, as suggested by the normative ISO527-1 [17], to a strain rate of 1% min^−1^, a speed test of 0.25 mm/min (tolerance ±20) was adopted and the number of 6 specimens per material and condition were tested by using the above-mentioned Instron Machine. With this testing setup, the elastic modulus and yield stress were characterized. Furthermore, their arithmetic mean value and standard deviation (for each material and condition) were determined and compared.

## 3. Results

Table 4 shows the results for elastic modulus and tensile yield stress of the materials analyzed under the different testing conditions, namely, as supplied (A.S.), thermoformed (T.) and stored in artificial saliva (S.A.S.), compared where possible to data sheet and supplier specification (D.S.).

The data sets relative to the mechanical properties were analyzed to verify if a significant statistical difference between the investigated test conditions occurred. The two-sample t-test (α = 0.05) was used and the null hypothesis for each test means that there is no significant difference for the mechanical properties considered (E and σ_y_). The null hypothesis was rejected for the elastic modulus of Duran and significant differences were observed for any test condition (A.S. vs. T., T. vs. S.A.S., A.S. vs. S.A.S., *p* < 0.05). The null hypothesis was also rejected for the elastic modulus of Zendura, except for A.S. vs. S.A.S. test conditions. The elastic modulus of Biolon, instead, showed no significant differences for all the test conditions (A.S. vs. T., T. vs. S.A.S., A.S. vs. S.A.S., *p* > 0.05). For the values of tensile yield stress the differences are significant only in the case of A.S. vs. T. and A.S. vs. S.A.S. for Zendura (*p* > 0.05). For the other materials tested under all the test conditions, the *p*-value is over the value of 0.05, and the null hypothesis cannot be rejected. The *p*-value for each material, comparing the results of all the tested conditions are reported in Table 5.

**Figure 4:**
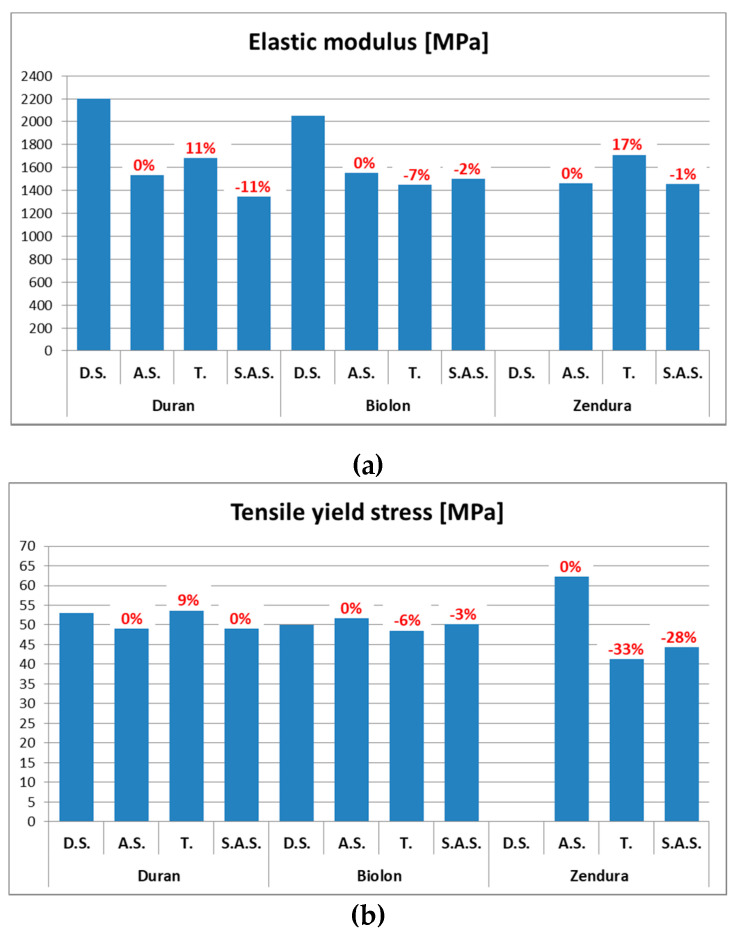
Mean value of elastic modulus (**a**) and tensile yield stress (**b**) for each material and test condition (D.S., A.S., T., S.A.S.). The percentage of increment or decrement after T. and S.A.S. with respect to A.S. is shown.

In light of the previous statistical considerations, the results (Table 4 and Figure 4) indicate how the thermoforming process (if compared to the A.S. condition) leads to a quite significant increase of the elastic modulus of Duran and Zendura (11% and 17% respectively). The same increase was not encountered for Biolon. The storage in artificial saliva did not show a considerable impact on the elastic modulus for Biolon. Conversely, a decrease of its value was found for Duran (11% if compared to A.S. and 19% if compared to T.) and Zendura, where a decrease of 15% was observed compared to T. The tensile yield stress, instead, is quite stable for Biolon and only small changes can be observed after T. and S.A.S. It increases, instead, after T. for Duran (9%) and significantly decreases for Zendura (33%) when compared to A.S. and decreased 28% after S.A.S. if compared again to A.S. Furthermore, where the data sheets (D.S.) were available (Duran and Biolon), the elastic modulus provided was much higher than the one experimentally derived by the tensile tests performed in the present study.

**Figure 5:**
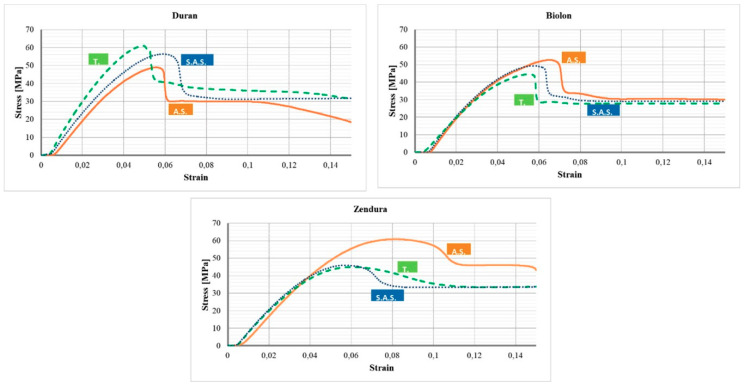
Representative stress-strain curves of Duran, Biolon and Zendura specimens: A.S., T., S.A.S.

Representative stress-strain curves are shown in Figure 5 to also show the mechanical behavior of the three materials tested, under each testing condition. By analyzing the stress-strain curves, the higher stiffness of Duran after T. is evident and, although the curve is representative of a single test and it is not a mean value, this observation is also in accordance with the higher mean value of the elastic modulus referred to Duran after T. reported in Table 4 and Figure 4. The stress-strain curves of Biolon, instead, show the stability of its mechanical behavior under different testing conditions and only a slight decrease of the stress at which plastic strain occurs can be noticed after T and S.A.S. Similarly, a decrease in the stress necessary to plastically strain the material, is also observed for Zendura, but in this case, it is quite significant. This is also in accordance with the results presented in Table 4 and Figure 4.

## 4. Discussion

The present study evaluated the effect of the thermoforming process, storage in artificial saliva and their combination on the mechanical properties of three thermoplastic polymers commonly used to fabricate clear aligners. In the literature, some interesting studies, which in part also inspired our work, were found. Eliades and Bourauel [21] in their review paper deepened the effect of storage in artificial saliva on chemical bond strength, due to the exposure of the thermoplastic polymers in the oral cavity, through the use of FT-IR chemical characterization. Bradley et al. [13] carried out a study on the same topic of Eliades and Bourauel [21] focusing on the comparison of chemical properties of Invisalign™ appliances before and after their use. Condò et al. [22] made, instead, an in-vitro study focused on the analysis of mechanical and chemico-physical properties of two different polymeric orthodontic aligners, Exceed30 and Smart Track, before and after use. In particular, they used a transversal approach relying on different characterization techniques (i.e., Fourier transform infrared spectroscopy, micro-Raman spectroscopy, X-ray diffraction, tensile and indentation strength test) [21]. Other studies, like the ones of Fang et al. [23] and of Lombardo et al. [24], with different approaches, investigated the stress relaxation of thermoplastic polymers used for orthodontics in temperature-regulated baths specifically designed to get a simulated oral environment. Ryu et al. [12], instead, focused their attention on the evaluation of the change in mechanical properties before and after thermoforming, with the aim to deepen the effect of the manufacturing process on the final mechanical behavior of the materials used. Our work also focuses on this aspect, but also analyzes the change in mechanical properties for specimens where thermoforming was combined with storage in a simulated intraoral environment. We deemed this combination necessary as an approach to get reliable data, and necessary to understand the final mechanical properties of the material used for the aligners and to design an effective and reliable orthodontic treatment. The results of the mechanical characterization, in fact, demonstrate that the manufacturing process and intraoral condition for some kinds of polymers can lead to changes in mechanical properties and that this effect can be different according to the specific material used. This finding suggests that a thermoplastic material to use in orthodontic appliances should be selected by characterizing its mechanical properties after its manufacturing process and its storage in a simulated intraoral environment. In particular, the results of the present study discussed in the previous section show that some of the polymers investigated are characterized by a higher elastic modulus and, thus, a higher stiffness after thermoforming and in one case (Duran) also a higher value of tensile yield stress. This behavior may be linked to a phenomenon called drawing, occurring for different polymers when pulled in tension (the condition of thermoforming: the polymer is heated and pulled in tension). In particular, the polymer chains slide over each other, unraveling, so that they become aligned with the direction of stretch. The drawn material is stronger and stiffer than before [25]. The results also show the impact of S.A.S. on the mechanical properties of the polymers. Elastic modulus and tensile yield stress of Biolon are quite stable and, as demonstrated also by the statistical differences discussed in the previous section, the changes caused by T. and S.A.S. are not particularly significant. The elastic modulus of Duran and Zendura, instead, are characterized by a significant decrease. A decrease can be observed also for the tensile yield stress of Duran (T. vs. S.A.S.) and Zendura (A.S. vs. S.A.S.). This decrease may be caused by the fluid absorption occurring during S.A.S. During S.A.S in fact the absorbed water may act as plasticizer, affecting and lowering the glass transition temperature (T_g_). The plasticizing effect of water can be a considerable issue in particular for amorphous polymers [26,27]. Properties such as elastic modulus have a linear temperature dependence, the closer the material is to T_g_, the lower the elastic modulus becomes [25].

Finally, considering clinical aspects, it is worth noting that fluid absorption could also affect the dimension of the aligner, compromising its fit on the patient arch. The combination of this aspect to the change in mechanical properties of the thermoplastic materials used can lead to a potential loss of efficacy due to the presence of unpredictable orthodontic force acting on teeth.

## 5. Conclusions

The main findings about the three thermoplastic materials studied are the following:

- the elastic modulus increases after thermoforming, except for Biolon^®^;
- the elastic modulus, compared to the material A.S., notably decreases after S.A.S. for Duran^®^. A slight decrease, instead, is observed in case of Biolon^®^ and Zendura^®^;
- the tensile yield stress increases after T. in the case of Duran^®^, slightly decreases in the case of Biolon^®^ and significantly decreases in the case of Zendura^®^ (from 62.37 MPa to 41.92 MPa);
- the tensile yield stress after S.A.S., compared to A.S., slightly increases for Duran^®^, decreases for Biolon^®^ and significantly decreases for Zendura^®^, but it is higher than after T.

The findings of the present study underline the necessity to investigate the mechanical properties of the thermoplastic polymers used for clear aligner therapy, after their manufacturing process and their storage in a simulated intraoral environment. The results, in fact, demonstrated that the impact of both on the mechanical properties can vary according to the specific polymer used. In conclusion, to design reliable and effective orthodontic treatments, the polymers should be selected after their mechanical properties’ characterization under conditions that are able to simulate the real operating environment of the aligner.

## Figures and Tables

**Table 1:** Heating and cooling time adopted during the thermoforming process. The cooling was carried out at room temperature and at a pressure of 4 bar.

Material	Heating Time (s)	Cooling Time (s)
Duran	30	60
Biolon	40	50
Zendura	35	50

**Table 2:** Chemical composition of artificial saliva (pH = 6.5) used to recreate the biochemical environment of human saliva.

Compound	Content (g/L)
NaCl	0.6
KCL	0.72
CaCl_2_·2H_2_O	0.22
KH_2_PO_4_	0.68
Na_2_HPO_4_·12H_2_O	0.856
KSCN	0.06
NaHCO_3_	1.5
C₆H₈O₇	0.03

**Table 3:** Weight variation of material specimen due to fluid absorption, before and after storage in artificial saliva.

Material	Before Storage in Artificial Saliva (g)	After Storage in Artificial Saliva (g)	Variation %
Duran	0.1334	0.1339	0.375
Biolon	0.1255	0.1261	0.438
Zendura	0.0974	0.0981	0.719

**Table 4:** Results of tensile tests for elastic modulus *E* and tensile yield stress *σ_y_* (mean value and standard deviation STDEV).

Material	Testing Condition	E (MPa)	STDEV	σ_y_ (MPa)	STDEV
Duran	D.S.	2200	-	53	-
	A.S.	1531	41	49.29	0.45
	T.	1693	51	53.52	4.84
	S.A.S.	1368	35	49.49	1.76
Biolon	D.S.	2050	-	50	-
	A.S.	1556	48	52.10	1.49
	T.	1447	42	48.75	2.57
	S.A.S.	1519	62	50.62	2.88
Zendura	D.S.	-	-	-	-
	A.S.	1478	88	62.37	0.90
	T.	1730	77	41.92	2.94
	S.A.S.	1466	72	44.61	1.82

**Table 5:** Results of t-test for all the materials and conditions tested.

Material	Conditions	*p*-Value, E	*p*-Value, σ_y_
Duran	A.S. vs. T.	0.0026	0.1325
	T. vs. S.A.S.	0	0.2355
	A.S. vs. S.A.S.	0.0009	0.8285
Biolon	A.S. vs. T.	0.0886	0.0790
	T. vs. S.A.S.	0.4107	0.4155
	A.S. vs. S.A.S.	0.3729	0.3957
Zendura	A.S. vs. T.	0.0013	0.0001
	T. vs. S.A.S.	0.0005	0.1706
	A.S. vs. S.A.S.	0.8220	0

## References

[1] Bucci R., Rongo R., Zito E., Valletta R., Michelotti A., D’Antò V. (2017). Translation and validation of the italian version of the Psychosocial Impact of Dental Aesthetics Questionnaire (pidaq) among adolescents. Eur. J. Paediatr. Dent..

[2] Bucci R., Rongo R., Zito E., Galeotti A., Valletta R., D’Antò V. (2014). Cross-cultural adaptation and validation of the Italian Psychosocial Impact of Dental Aesthetics Questionnaire (PIDAQ). Qual. Life Res..

[3] Miller K.B., McGorray S.P., Womack R., Quintero J.C., Perelmuter M., Gibson J., Dolan T.A., Wheeler T.T. (2007). A comparison of treatment impacts between Invisalign aligner and fixed appliance therapy during the first week of treatment. Am. J. Orthod. Dentofac. Orthop..

[4] Rossini G., Parrini S., Castroflorio T. (2015). Efficacy of Clear Aligners in Controlling Orthodontic Tooth Movement: A Systematic Review. Smile Dent. J..

[5] Papadimitriou A., Mousoulea S., Gkantidis N., Kloukos D. (2018). Clinical effectiveness of Invisalign (R) orthodontic treatment: A systematic review. Prog. Orthod..

[6] Cervinara F., Cianci C., De Cillis F., Pappalettera G., Pappalettere C., Siciliani G., Lombardo L. (2019). Experimental Study of the Pressures and Points of Application of the Forces Exerted between Aligner and Tooth. Nanomaterials.

[7] Kohda N., Iijima M., Muguruma T., A Brantley W., Ahluwalia K.S., Mizoguchi I. (2012). Effects of mechanical properties of thermoplastic materials on the initial force of thermoplastic appliances. Angle Orthod..

[8] Savignano R., Viecilli R.F., Paoli A., Razionale A., Barone S. (2016). Nonlinear dependency of tooth movement on force system directions. Am. J. Orthod. Dentofac. Orthop..

[9] Barone S., Paoli A., Razionale A. (2013). Computer-aided modelling of three-dimensional maxillofacial tissues through multi-modal imaging. Proc. Inst. Mech. Eng. Part H.

[10] Barone S., Paoli A., Razionale A. (2013). Multiple alignments of range maps by active stereo imaging and global marker framing. Opt. Lasers Eng..

[11] Alexandropoulos A., Al Jabbari Y.S., Zinelis S., Eliades T. (2015). Chemical and mechanical characteristics of contemporary thermoplastic orthodontic materials. Aust. Orthod. J..

[12] Ryu J.-H., Kwon J.-S., Jiang H.B., Cha J.-Y., Kim K.-M. (2018). Effects of thermoforming on the physical and mechanical properties of thermoplastic materials for transparent orthodontic aligners. Korean J. Orthod..

[13] Bradley T.G., Teske L., Eliades T., Zinelis S. (2015). Do the mechanical and chemical properties of Invisalign TM appliances change after use? A retrieval analysis. Eur. J. Orthod..

[14] Ma Y.S., Fang D.Y., Zhang N., Ding X.J., Zhang K.Y., Bai Y.X. (2016). Mechanical Properties of Orthodontic Thermoplastics PETG/ PC2858 after Blending. Chin. J. Dent. Res..

[15] Barone S., Paoli A., Neri P., Razionale A., Giannese M. (2016). Mechanical and Geometrical Properties Assessment of Thermoplastic Materials for Biomedical Application. Advances on Mechanics, Design Engineering and Manufacturing.

[16] Papadopoulou A.K., Cantele A., Polychronis G., Zinelis S., Eliades T. (2019). Changes in Roughness and Mechanical Properties of Invisalign((R)) Appliances after One- and Two-Weeks Use. Materials.

[17] Standardization IOf (2012). ISO 527-1:2012 Plastics–Determination of Tensile Properties.

[18] Walele S.P.A., Chaudhari A., Patil C., Yaragamblimath P., Survase R. (2016). Leaching from Thermoplastic Sheets-A Quantitative Assessment. Int. J. Contemp. Med Res..

[19] Duffo G.S., Quezada-Castillo E. (2004). Development of an Artificial Saliva Solution for Studying the Corrosion Behavior of Dental Alloys. Corrosion.

[20] Porcayo-Calderon J., Casales-Diaz M., Salinas-Bravo V.M., Martinez-Gomez L. (2015). Corrosion Performance of Fe-Cr-Ni Alloys in Artificial Saliva and Mouthwash Solution. Bioinorg. Chem. Appl..

[21] Eliades T., Bourauel C. (2005). Intraoral aging of orthodontic materials: The picture we miss and its clinical relevance. Am. J. Orthod. Dentofac. Orthop..

[22] Condo’ R., Pazzini L., Cerroni L., Pasquantonio G., Lagana’ G., Pecora A., Mussi V., Rinaldi A., Mecheri B., Licoccia S. (2018). Mechanical properties of “two generations” of teeth aligners: Change analysis during oral permanence. Dent. Mater. J..

[23] Fang D., Zhang N., Chen H., Bai Y. (2013). Dynamic stress relaxation of orthodontic thermoplastic materials in a simulated oral environment. Dent. Mater. J..

[24] Lombardo L., Martines E., Mazzanti V., Arreghini A., Mollica F., Siciliani G. (2017). Stress relaxation properties of four orthodontic aligner materials: A 24-hour in vitro study. Angle Orthod..

[25] Ashby M.F. (2018). Hugh Shercliff, and David Cebon, Materials: Engineering, Science, Processing and Design.

[26] Yang B., Huang W., Li C., Li L. (2006). Effects of moisture on the thermomechanical properties of a polyurethane shape memory polymer. Polymer.

[27] Hancock B., Zografi G. (1994). The Relationship Between the Glass Transition Temperature and the Water Content of Amorphous Pharmaceutical Solids. Pharm. Res..

